# EXAMINING THE ASSOCIATION BETWEEN ACTIVE COPING, GRIT, AND SUBJECTIVE COGNITIVE DECLINE IN AGING BLACK AMERICAN MEN

**DOI:** 10.1093/geroni/igae098.1597

**Published:** 2024-12-31

**Authors:** Candi Nwakasi, Darlingtina Esiaka

**Affiliations:** University of Connecticut, Storrs, Connecticut, United States; University of Kentucky College of Medicine, University of Kentucky, Kentucky, United States

## Abstract

**Background:**

Active coping and grit have been associated with positive health outcomes including cognitive functioning. However, to the best of our knowledge, no previous study has examined the effect of active coping and grit on subjective cognitive decline among Black American men. Further, this study’s focus on subjective cognitive decline (SCD) in aging Black men is crucial because SCD may indicate an onset of Alzheimer’s disease and related dementia and/or other brain health issues.

**Methods:**

Participants (N=138, Mage = 48) who identified as Black American men living in urban areas responded to an online survey measuring sociodemographic factors, grit, active coping, and subjective cognitive decline. We used hierarchical regression in the analysis to examine the association between active coping, grit, and subjective cognitive decline.

**Results:**

Analysis revealed that active coping (B = -0.196, p=.037) and grit (B = 0.643, p<.001) were significant predictors of SCD while controlling for demographic variables (F [7,130] = 7.753, p<0.001). Also, moderation analysis showed a significant interaction between active coping and grit on SCD (β = -0.04, SE = 0.02, 95%CI [0.00,0.08], p <.041).

**Conclusion:**

By focusing specifically on the association of active coping, grit, and SCD, this study offers insight on the unique experiences of Black American men and contribute to a more nuanced understanding of their cognitive well-being. Thus, the results may inform targeted interventions and support systems for aging Black men’s brain health.

